# Early Steps in C-Type Inactivation of the hERG Potassium Channel

**DOI:** 10.1021/acs.jcim.2c01028

**Published:** 2022-12-13

**Authors:** Francesco Pettini, Carmen Domene, Simone Furini

**Affiliations:** † Department of Medical Biotechnologies, University of Siena, viale Mario Bracci 12, Siena 53100, Italy; ‡ Department of Biotechnology, Chemistry and Pharmacy, University of Siena, viale Mario Bracci 12, Siena 53100, Italy; § Department of Chemistry, University of Bath, Claverton Down, Bath BA2 7AY, U.K.; ∥ Department of Chemistry, University of Oxford, Mansfield Road, Oxford OX1 3TA, U.K.; ⊥ Department of Electrical, Electronic and Information Engineering ″Guglielmo Marconi”, University of Bologna, via dell’Università 50, Cesena (FC) 47521, Italy

## Abstract

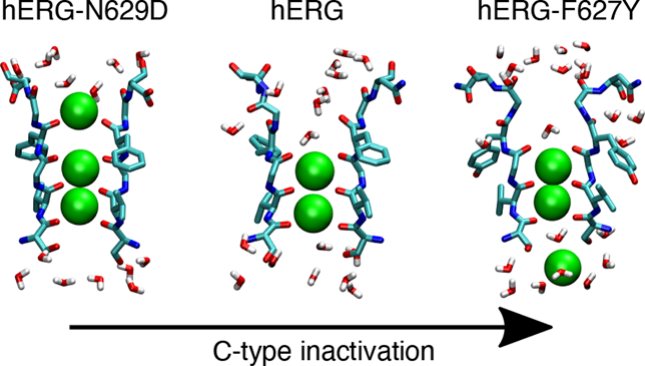

Fast C-type inactivation confers distinctive functional properties to the hERG potassium channel, and its association to inherited and acquired cardiac arrythmias makes the study of the inactivation mechanism of hERG at the atomic detail of paramount importance. At present, two models have been proposed to describe C-type inactivation in K^+^-channels. Experimental data and computational work on the bacterial KcsA channel support the hypothesis that C-type inactivation results from a closure of the selectivity filter that sterically impedes ion conduction. Alternatively, recent experimental structures of a mutated Shaker channel revealed a widening of the extracellular portion of the selectivity filter, which might diminish conductance by interfering with the mechanism of ion permeation. Here, we performed molecular dynamics simulations of the wild-type hERG, a non-inactivating mutant (hERG-N629D), and a mutant that inactivates faster than the wild-type channel (hERG-F627Y) to find out which and if any of the two reported C-type inactivation mechanisms applies to hERG. Closure events of the selectivity filter were not observed in any of the simulated trajectories but instead, the extracellular section of the selectivity filter deviated from the canonical conductive structure of potassium channels. The degree of widening of the potassium binding sites at the extracellular entrance of the channel was directly related to the degree of inactivation with hERG-F627Y > wild-type hERG > hERG-N629D. These findings support the hypothesis that C-type inactivation in hERG entails a widening of the extracellular entrance of the channel rather than a closure of the selectivity filter.

## Introduction

C-type inactivation is a property of K^+^-channels, which refers to a decrease in their conductance under a sustained gating stimulus, mediated by structural changes in the extracellular region of the pore and sensitive to the extracellular concentration of potassium ions.^1^ In the particular case of the voltage-gated hERG channel, the rate of C-type inactivation is in the millisecond range.^2^ As a result of this fast inactivation, combined with slow gating, the conductance of hERG peaks during repolarization of the membrane potential following a depolarizing impulse.^3^ These unusual conduction properties make hERG a crucial player during the repolarization phase of action potentials in cardiac cells. Therefore, not surprisingly, alterations of hERG currents are responsible for inherited^4^ or acquired^5^ cardiac arrythmias. In this respect, understanding the atomic details of C-type inactivation is crucial to disclose the mechanisms underneath the hereditary diseases resulting from hERG mutations and to provide useful information about the pharmacological properties of this channel, with potential implications on drug discovery.

**Figure 1:**
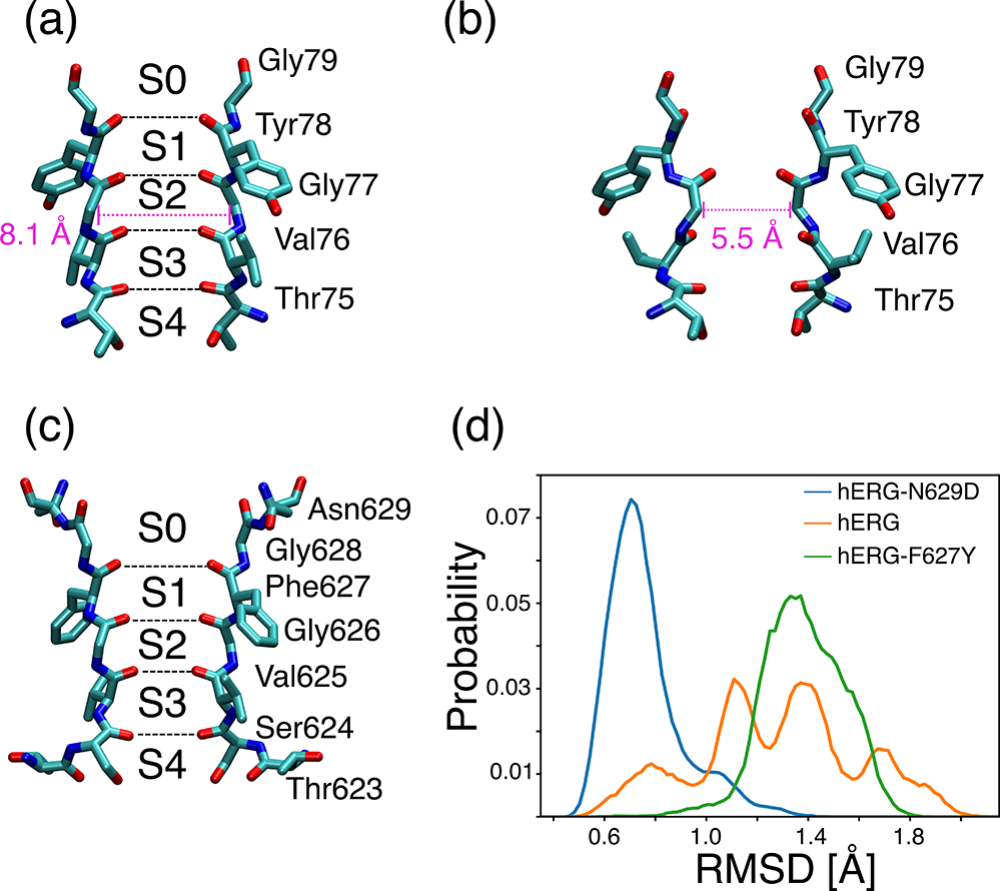
Experimental (a) conductive and (b) closed structures of the KcsA selectivity filter (SF). Residues Thr75 to Gly79 of two opposite subunits from PDBs 1K4C and 1K4D are shown in licorice representation. Labels indicating the position of binding sites S0–S4 are shown in the conductive structure. Distances between opposite Cα atoms of residues Gly77 are shown in pink. (c) Experimental structure of the hERG SF. Residues Thr623 to Asn629 of two opposite subunits from PDB 5VA2 are shown in licorice representation, with labels indicating the position of binding sites S0–S4. (d) Root-mean-square deviation (RMSD) of the SF in simulations of hERG-N629D, wild-type hERG, and hERG-F627Y. The RMSD is defined as the distance of the backbone atoms of residues Thr623 to Asn629 from the corresponding atoms in the conductive structure of KcsA (PDB 1K4C). The RMSD distributions are estimated using cumulative data from eight independent 1 μs trajectories for each model system.

Most of the current knowledge about the atomic mechanisms of C-type inactivation has resulted from the studies of the bacterial KcsA potassium channel. When KcsA is crystallized in a high-K^+^ concentration, the region responsible for selective conduction of potassium ions, the so-called selectivity filter (SF), presents five K^+^ binding sites, named S0–S4 starting from the extracellular side (Figure 1a).^6^ In contrast, in a low-K^+^ concentration, the structure of the SF assumes an hour-glass shape that is constricted at binding site S2 (Figure 1b).^6^ Similar closed structures of the SF were also observed when KcsA was crystallized with the intracellular gate in the open state.^7^ Since KcsA exhibits an inactivation mechanism resembling the characteristics of C-type inactivation in voltage-gated channels, the closed structure of the KcsA SF was immediately associated with the C-type inactivated state. Molecular dynamics (MD) simulations based on the experimental structures of KcsA have revealed the role of protein residues surrounding the SF as well as nearby water molecules on the stability of the conductive and closed structures of the SF,^8,9^ providing a coherent framework for the interpretation of experimental data on channel inactivation. Based on experimental and computational studies, the current accepted model of C-type inactivation in KcsA suggests that intracellular gate opening is allosterically transmitted to the SF, causing closure of its central region and impeding ion conduction.

The link between the closed structure of the SF and C-type inactivation is yet to be experimentally confirmed in ion channels other than KcsA. Remarkably, the closed structure of the SF has been experimentally reported only in KcsA, and in particular, at odds with its fast inactivation properties, the experimental structure of the SF in hERG is indistinguishable from conductive structures previously observed in KcsA and other K^+^-channels (Figure 1c).^10^ This apparent disagreement between the conductive structure of the SF and the expected inactivated state of the channel might reflect the fact that the conditions adopted in structural determination experiments could perturb the delicate equilibrium between different structures of the SF. In this respect, using MD simulations is a powerful strategy to complement the experimental work and to investigate the dynamics of the SF at atomic details. Previously reported MD simulations starting from the experimental structure of hERG showed that the SF evolves toward a closed structure, similar to the closed structure experimentally observed in KcsA, over a time period as fast as hundreds of nanoseconds.^11,12^ Analogous fast closure events of the SF were also observed in MD simulations of the Shaker K^+^-channel, another voltage-gated channel that undergoes C-type inactivation.^13^ These observations of spontaneous transitions toward the closed structure of the SF in simulations of inactivating channels support the hypothesis that the closure of the SF is indeed responsible for C-type inactivation, not only in KcsA but also in voltage-gated potassium channels.

The link between C-type inactivation and closure events of the SF observed in MD simulations becomes less obvious when the effect of the force field employed on the stability of the conductive structure of the channel is considered. In a previous study, we showed that the conductive SF spontaneously evolves toward a closed structure in the sub-millisecond timescale in simulations with the CHARMM force field, even if channels that do not undergo inactivation are considered.^14^ To the best of our knowledge, all the MD simulations reported in the literature that describe gating events at the SF of K^+^-channels, including hERG, were performed using the CHARMM force field. Therefore, to reach robust conclusions about hERG inactivation, MD simulations with different force fields are necessary. Here, MD simulations of wild-type hERG and of two mutants with altered inactivation properties, hERG-N629D and hERG-F627Y, are reported using the AMBER force field. hERG-N629D is a non-inactivating channel,^15^ while hERG-F627Y inactivates faster and to a higher degree than the wild-type hERG.^16^ None of the MD simulations considered in this study revealed any closure event of the SF. Instead, a direct relationship between the extent of structural changes in the extracellular portion of the SF and the strength of C-type inactivation was observed.

## Methods

### MD Simulations

The atomic model of the hERG channel was based on the Protein Data Bank (PDB) entry 5VA2.^10^ Only the pore region of the channel was included in the model, from residue Tyr545 to residue Tyr667. MODELLER was used to build the initial positions of missing residues in the extracellular loop regions: His578 to Arg582 and Asn598 to Leu602.^17^ The models of hERG-N629D and hERG-F627Y were built by manually replacing the mutated residues in the initial wild-type model. The simulation systems were built using CHARMM-GUI.^18^ The lipid membrane was composed by a mixture of 1-palmitoyl-2-oleoyl-glycero-3-phosphocholine (POPC) and 1-palmitoyl-2-oleoyl-sn-glycero-3-phosphate (POPA) in a 3-POPC:1-POPA ratio. The pore axis was aligned to the *z*-axis of the simulation box. The system was solvated using TIP3P water molecules^19^ (∼17,000 molecules), and 200 mM KCl was added to neutralize the system. Potassium ions were manually placed at binding sites S0, S2, and S4 in the SF. The ff14sb version of the AMBER force field was used,^20^ in combination with ion parameters by Joung and Cheatham^21^ and the TIP3P water model.^19^ Van der Waals interactions were truncated at 9 Å. A standard AMBER scaling of 1–4 interactions was applied. Long-range electrostatic interactions were calculated with the particle mesh Ewald method using a grid spacing of 1.0 Å.^22^ The SETTLE algorithm was used to restrain bonds with hydrogen atoms.^23^ The temperature was controlled at 310 K by coupling to a Langevin thermostat with a damping coefficient of 1 ps^–1^. A pressure of 1 atm was maintained by coupling the system to a Nosé–Hoover Langevin piston, with a damping constant of 25 ps and a period of 50 ps.^24^ NAMD2.12 was used for all the simulations.^25^ The equilibration protocol consisted of 10,000 steps of energy minimization, followed by 15 ns of dynamics in the NPT ensemble with a 1-fs timestep and 70 ns in the NPT ensemble with a 2-fs timestep. In the course of the equilibration, restraints on protein and lipid atoms were gradually reduced to zero. Subsequently, eight independent trajectories of 1 μs each were simulated for each of the three models. Atomic coordinates were saved every 10 ps. The atomic model of the KcsA channel was based on the experimental structure in the open/conductive state, PDB entry 5VK6.^26^ The entire transmembrane domain of KcsA, from residue Trp26 to residue Gln121, was considered. Residue Ala71 was manually mutated to glutamate as in the wild-type (inactivating) KcsA channel. The same protocol described above for hERG was used to define the initial coordinates of the KcsA model system and for the equilibration. In order to prepare the KcsA atomic model with the SF in the closed state, ions were removed from binding sites S0–S4, and harmonic restraints were applied to backbone atoms of the SF in the course of a 10 ns simulation using as a reference PDB entry 1K4D.^6^ Subsequently, harmonic restraints were removed, and four independent trajectories of 1 μs each were simulated.

### Analyses of the MD Trajectories

The presence of potassium ions in binding sites S0–S4 was evaluated by the following equation:
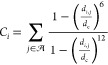
1which calculates the coordination number, *C_i_*, at the position defined by index *i* from contributions by atoms in selection . In eq 1, *d*_*i*, *j*_ is the distance between atom *j* and the position defined by index *i* and *d*_c_ is a cut-off distance after which the contribution of atom *j* to the coordination number decreases to zero as dictated by the 6, 12 exponents. When eq 1 was used to estimate the occupancy of binding sites by K^+^, all the potassium ions in the system were considered in ; the index *i* identified the center of the binding site, *i* ∈ {S0, S1, S2, S3, S4}, defined as the average position of the eight oxygen atoms delineating the site, and the cut-off distance was assumed equal to 1.4 Å, which corresponds to approximately half of the binding site length along the channel axis. In this way, the coordination number tends to 1 when a K^+^ ion is close to the center of the corresponding binding site and it approaches 0 if a binding site is empty. The occupancy of binding sites S0–S4 by K^+^ as estimated by eq 1 was used to cluster the MD trajectories. Clustering was performed separately for each simulated system using the agglomerative clustering algorithm with the Euclidean distance and the Ward linkage criterion.^27^ The maximum of the silhouette score was used to identify the optimal number of clusters. The atomic structures assigned to the various clusters were further evaluated by considering the coordination number of oxygen atoms in binding sites S0–S4 and the presence of K^+^ and water molecules in the intracellular cavity. The coordination number of oxygen atoms in binding sites S0–S4 was estimated using eq 1, as before, but with the set of atoms  corresponding to any oxygen atom in the system and a cut-off distance *d*_c_ equal to 3.2 Å, which is similar to the radius of the first hydration shell of a potassium ion. The same approach was used to characterize the properties of the intracellular cavity. To estimate the presence of K^+^ in the intracellular cavity, first the value of *C_i_* was calculated, using a set  that included all potassium ions, and setting *d*_c_ to 1.4 Å, for a set of equally spaced points along the axis of the channel, starting from a position 2 Å below the lower boundary of S4 in the intracellular direction, and extending 20 Å toward the intracellular compartment. Then, the maximum among the values of the coordination number along the axis of the channel was taken as an estimate of the presence of K^+^ in the cavity. The advantage of using the maximum value is that it is not affected by the *z*-coordinate of potassium ions along the channel axis, and consequently, it evaluates the presence of K^+^ in the cavity regardless of the exact ion positions. The presence of water molecules in the intracellular cavity was estimated as the minimum of eq 1 along the axis of the channel, including in  all the oxygen atoms and setting *d*_c_ to 3.2 Å, as previously done for estimating the oxygen coordination number in binding sites S0–S4. In this case, the use of the minimum value is motivated by the need to identify possible regions with lack of hydration along the axis of the channel, which might prevent ion conduction. Trajectory analyses were performed with custom python code using MDanalysis^28^ and the Scipy ecosystem.^29^ VMD was used to inspect trajectories and to generate images of the systems.^30^

## Results

The aim of this study is to analyze the dynamics of the SF in hERG and in two mutated hERG channels with altered inactivation properties, hERG-N629D and hERG-F627Y, to get some insights into the atomic details of C-type inactivation. Since C-type inactivation has been associated with closure of the SF, we first evaluated the radius of the SF in the atomic trajectories of the three model systems. In the KcsA channel, the distance between Cα atoms of residues Gly77 is 8.1 Å in the conductive structure (PDB code: 1K4C) and 5.5 Å in the closed structure (PDB code: 1K4D). The distance between opposing subunits of the corresponding residues in hERG (Gly626) was 7.9 ± 0.3 Å, 8.1 ± 0.2 Å, and 7.9 ± 0.3 Å in simulations of the wild-type channel, hERG-N629D, and hERG-F627Y respectively. In all the simulated trajectories (eight for each model system), the average radius is higher than 7.8 Å, meaning that not a single closure event of the SF took place. The lack of closure events in these MD simulations using the AMBER force field, compared to previous simulations reported in the literature^11,12^ using the CHARMM force field, might reflect the inability of the AMBER force filed to sample the occluded state of the SF. To test this hypothesis, simulations of the wild-type KcsA channel were performed starting from an equilibrated system with the SF in the closed state and no ions inside. These simulations showed that the closed structure of the SF is stable in microsecond trajectories using the AMBER force field (Figure S1 in Supporting Information).

To further characterize the structure of the SF in simulations of the hERG channel, the average distances between opposite carbonyl oxygen atoms at the boundaries of binding sites S0–S4 were measured (Table 1). In simulations of hERG-N629D, the distances between opposite carbonyl oxygen atoms in the SF agree with the analogous values measured in the experimental conductive structure of KcsA. In contrast, in both wild-type hERG and hERG-F627Y, the boundary between binding sites S1 and S0 (carbonyl oxygen atoms of Phe627 in wild-type hERG and of Tyr627 in hERG-F627Y) is almost 4 Å wider than in the conductive structure. In agreement with these atomic distances, the RMSD of the backbone atoms of the SF from the corresponding backbone atoms in the conductive structure of the KcsA channel (PDB code: 1K4C) was found to be significantly different among the three model systems (Figure 1d). In MD simulations of hERG-N629D, the conductive structure of the SF is well-preserved with an average RMSD of 0.7 Å. Higher RMSD values were observed for both the wild-type hERG and the hERG-F627Y models. In the wild-type channel, distinct peaks appear in the distribution of RMSD values, which suggests that the SF samples alternative configurations, some of which are close to the canonical conductive structure. In contrast, in hERG-F627Y, a single peak distribution of RMSD values is centered around 1.3 Å, which indicates that the SF deviates from the conductive structure in all the simulated trajectories. The deviations of the SF from the conductive structure emerging from RMSD distributions qualitatively agree with the degree of inactivation in the three systems considered.

**Table 1:** Average Distance in Ångström between Carbonyl Oxygen Atoms in Opposite Subunits for Residues 624–627^a^

	hERG-N629D	wild-type hERG	hERG-F627Y	KcsA
[F/Y]627	5.8 ± 1.1	8.9 ± 1.8	9.2 ± 1.1	5.1
G626	5.1 ± 0.8	5.3 ± 1.0	5.2 ± 0.7	4.7
V625	4.7 ± 03	5.0 ± 0.8	4.7 ± 0.3	4.7
S624	4.8 ± 0.3	4.9 ± 0.4	4.5 ± 0.3	4.5

a Average values and standard deviations were computed using eight 1-μs independent trajectories for each of the three model systems. The selected oxygen atoms are the ones that define the boundaries between pairs of successive binding sites for K^+^ in the SF, as indicated by dashed black lines in Figure 1a,c. The equivalent KcsA distances refer to residues T75 to Y78 in PDB 1K4C.

**Figure 2:**
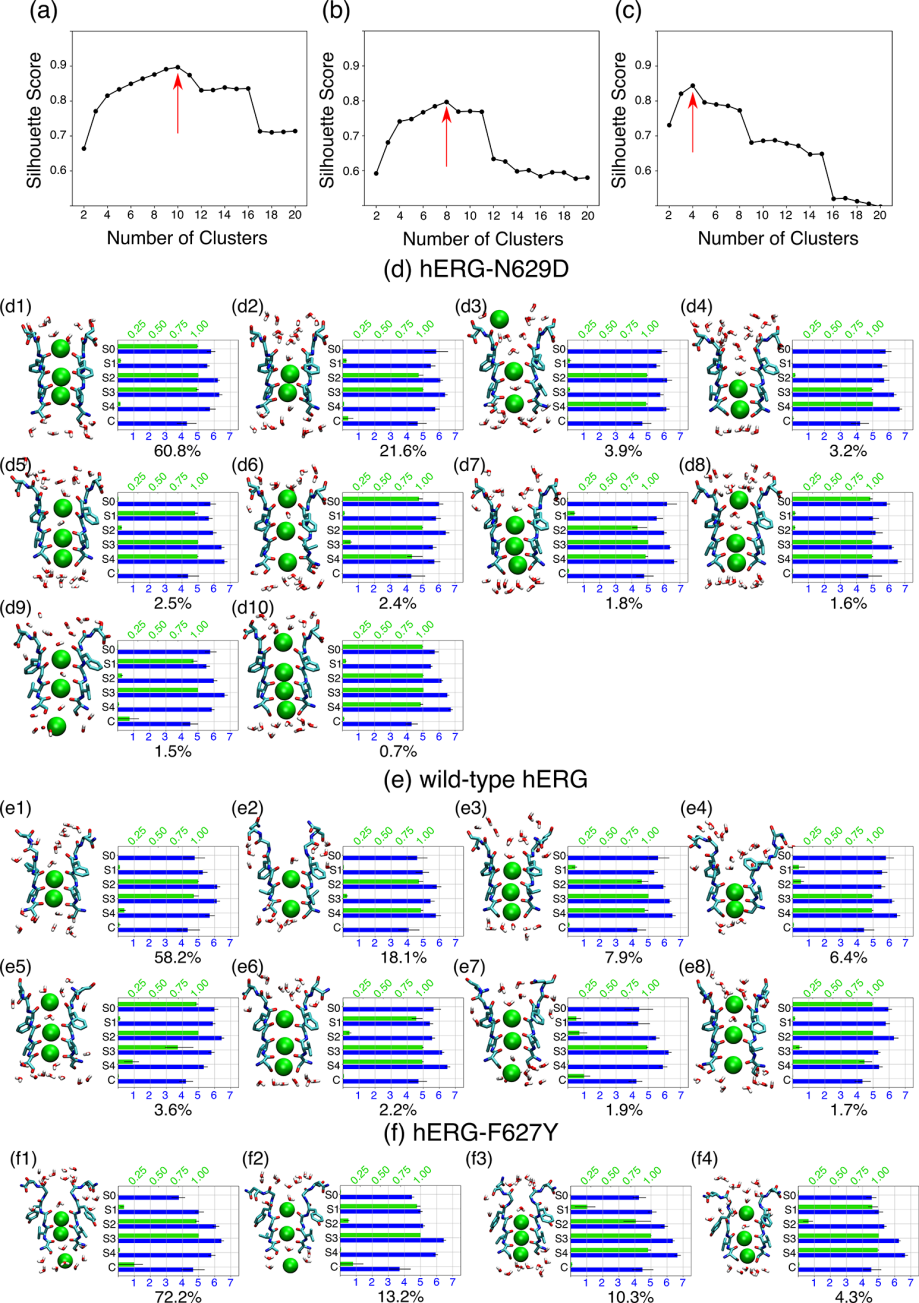
The silhouette score as a function of the number of clusters is shown for hERG-N629D (a), wild-type hERG (b), and hERG-F627Y (c). The red arrow indicates the maxima of the silhouette score used to define the optimal number of clusters. Clustering of the SF in hERG-N629D (d), wild-type hERG (e), and hERG-F627Y (f). The K^+^ occupancy of binding sites S0–S4 and the cavity is represented by green bars. The number of oxygen atoms contributed from either the protein or water molecules at binding sites S0–S4, and the cavity is represented by blue bars. For green and blue bars, black lines indicate the first and third quartiles of the corresponding features in the cluster. The probability of each cluster is reported below each plot. A representative snapshot of each cluster is shown illustrating residues 624–629 and water molecules closer than 3 Å to the protein oxygen atoms defining binding sites S0–S4 in licorice representation. K^+^ ions are shown as green spheres.

To better identify the ensemble of structures sampled by the SF, the trajectories were clustered considering the ion occupancy of binding sites S0–S4. The use of ion configurations as clustering features is motivated by the direct link between the presence of ions at particular binding sites and the structure of the SF (e.g., an ion in S2 clearly prevents the closed structure of the SF experimentally observed in KcsA). In addition, clustering trajectories using ion configurations render states that are easier to interpret because, for instance, they correspond to common configurations observed in conduction events. For each model system, the maximum of the silhouette score was used to define the optimal number of clusters. The silhouette score estimates how samples within clusters are similar compared to how clusters are different from each other, and consequently, it is maximum for the clustering scheme that provides the best separation among homogeneous clusters. Here, we found that the optimal number of clusters was 10, 8, and 4, respectively, for hERG-N629D, wild-type hERG, and hERG-F627Y (Figure 2a–c). In other words, more alternative configurations of ions in the SF are recorded in wild-type hERG and in hERG-N629D than in hERG-F627Y. The set of ion configurations explored by wild-type hERG and hERG-N629D resembles the one described in MD simulations of other ion channels.^31−34^ Specifically, in both systems, ions are found in contiguous binding sites S2 and S3 in the most populated cluster. The difference between hERG-N629D and wild-type hERG is the higher ion probability in S0 in the former, which can be explained by the presence of a ring of negative charges contributed by the aspartate residues (Asp629) at the outer entrance of the SF. Together with the different ion configurations and associated probabilities, the most obvious difference between hERG-N629D and the wild-type channel is the geometry of binding sites S0 and S1. The structural integrity of binding sites S0–S4 was evaluated by estimating the number of oxygen atoms at a 3.2 Å distance from the binding site centers. In hERG-N629D, binding sites S0–S4 are characterized by more than five oxygen atoms in all the clusters, in agreement with previous observations in other K^+^-channels in the conductive state.^35^ In contrast, in clusters e1, e2, and e7 of the wild-type channel (Figure 2), the average number of oxygen atoms is lower than 5. This reflects widening of binding sites S0 and S1, in line with the distances between carbonyl oxygen atoms at the boundary between these two binding sites reported in Table 1. The loss of oxygen atoms at a 3.2 Å distance from the center of binding sites S0 and S1 observed in clusters e1, e2, and e7 of the wild-type channel characterizes all the clusters of hERG-F627Y. In this mutated channel with enhanced C-type inactivation, the conductive structure of binding sites S0 and S1 was not sampled in any of the eight 1 μs independent trajectories. The loss of binding sites S0 and S1 impacts the number of accessible ion configurations, which explains the lower number of optimal clusters estimated for hERG-F627Y compared to those for wild-type hERG and hERG-N629D.

**Figure 3:**
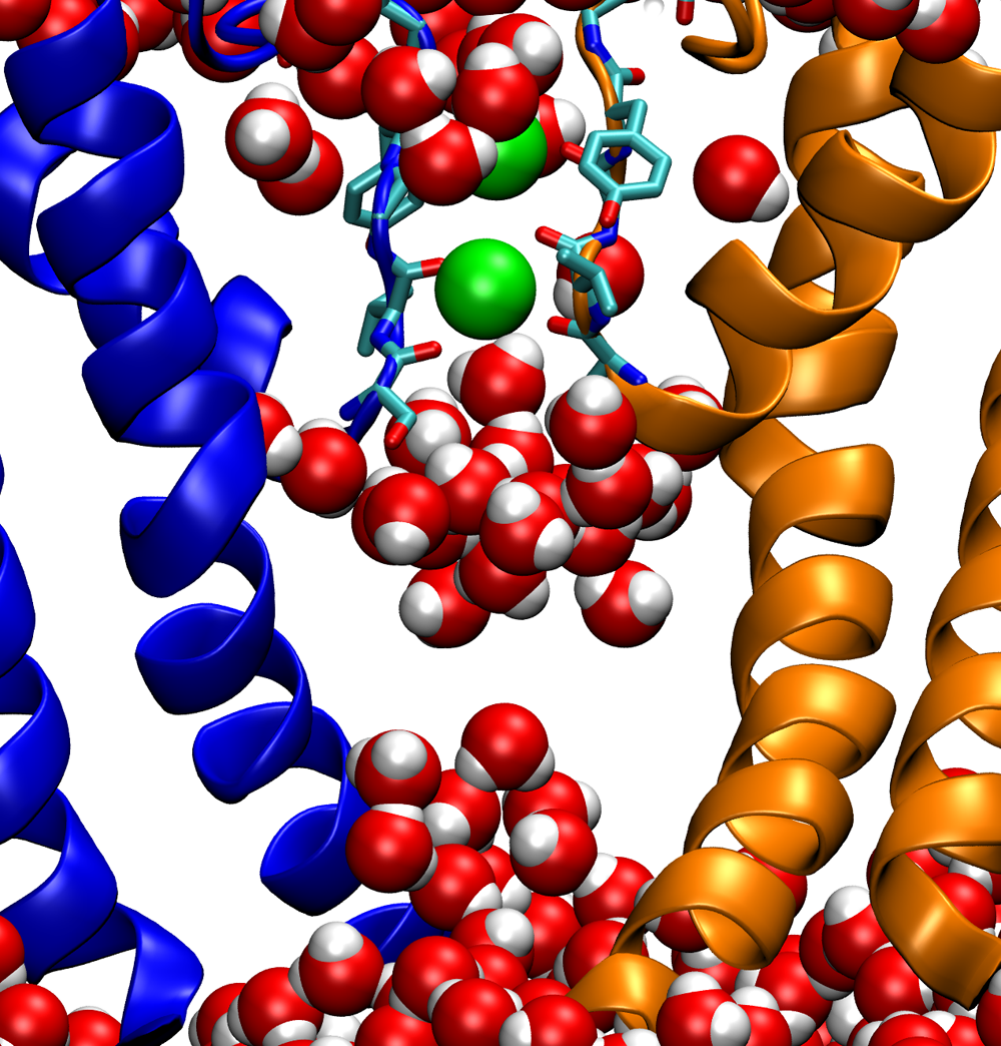
Lack of continuous hydration in the channel cavity of hERG-F627Y. Representative snapshot from cluster f2 of Figure 2 where a gap in hydration is observed at the center of the cavity. Only two opposite subunits in cartoon representation in orange and blue are shown for clarity. Residues of the SF are shown in licorice representation. K^+^ ions (green) and water molecules are shown in VdW representation.

The different distribution of ions in the three model systems might be linked to differences in the occupancy of the intracellular cavity by ions and water molecules. Therefore, the presence of ions and water molecules in the intracellular cavity was also evaluated (labels “C” in Figure 2d–f). Stable binding (>10 ns) of potassium ions in the intracellular cavity was not observed for any of the model systems. In the few clusters where the average ion occupancy of the cavity was close to 1, K^+^ ions were more likely to be observed in the upper region of the cavity, closer to binding site S4 (clusters d9, e7, f1, and f2 in Figure 2) than to the cavity center. The absence of a stable K^+^ binding site in the intracellular cavity might be related to its low internal volume (Figures S2–S4 in Supporting Information). The most constricted region of the cavity was in the neighborhood of residues Tyr652 in all the systems. The number of coordinating oxygen atoms in this constricted region of the cavity (blue bars labeled “C” in Figure 2d–f) ranges from 4 to 5 in any cluster apart from cluster f2 of hERG-F627Y, where it drops below 4. Configurations that belong to this cluster correspond to channel structures with a partially dehydrated intracellular cavity, as exemplified by a representative snapshot in Figure 3. A low number of water molecules in the cavity might also contribute to reduce the conductance of the channel.

## Discussion

The role of hERG in acquired and inherited cardiac arrythmias justifies the profound interest in characterizing the atomic mechanism of its C-type inactivation. In the currently available experimental atomic structures of hERG, the SF resembles the canonical conductive state previously observed in other potassium channels.^10^ Previous MD simulations revealed that the experimental structure of the SF of hERG spontaneously evolves toward a closed state in a sub-millisecond timescale,^11,12^ suggesting a link between gating of the SF and C-type inactivation. The results presented here advocate for a different mechanism of C-type inactivation in hERG. Closure events of the SF were not sampled in any of the three model systems, wild-type hERG, hERG-N629D, or hERG-F627Y, over a cumulative simulation time of 24 μs. Instead, widening of the K^+^ binding sites S0 and S1 at the extracellular entrance of the SF was observed. The extent of these structural changes runs in parallel to the degree of C-type inactivation, with hERG-F627Y > wild-type hERG > hERG-N629D. The main methodological difference between the simulations presented here and in previous studies is the adopted force field; AMBER was employed in this study and CHARMM in refs (11, 12). At this point, it is important to remember that differences between simulations using the AMBER and CHARMM force fields were reported in the context of the stability of the SF in other potassium channels;^14,36^ the conductive structure of the SF is not stable in the millisecond timescale in simulations using the CHARMM force field, regardless of the inactivation properties of the ion channel under investigation. In CHARMM-based simulations, the SF closes in microsecond trajectories even when non-inactivating channels are considered, and indeed, microsecond trajectories with the potassium channels in the conductive state were obtained only by adding ad hoc terms to the force field.^37^ These ad hoc terms are not needed in simulations using AMBER force fields, where the conductive state of the SF is stable. The stability of the SF in simulations with AMBER or CHARMM is not affected by the application of physiological membrane potentials.^14^

Despite this important difference between simulations using AMBER and CHARMM force fields, it is worth noting that in both cases, the trend between the strength of C-type inactivation and the departure of the SF from the canonical conductive structure is maintained.^11,12^ This qualitative agreement with experimental data strengthens the case of employing MD simulations as a complementary tool to experimental analyses to help reveal the details of C-type inactivation. What remains to be established is the extent and the rate of the structural changes of the SF in the hERG and other potassium channels undergoing C-type inactivation. In particular, in the case of the hERG channel, it should be established if C-type inactivation entails an almost instantaneous closure of the SF, as observed in MD simulations using the CHARMM force field, or if instead, C-type inactivation results from widening the extracellular portion of the SF and the consequent elimination of S0 and S1 binding sites, as observed in simulations using the AMBER force field.

**Figure 4:**
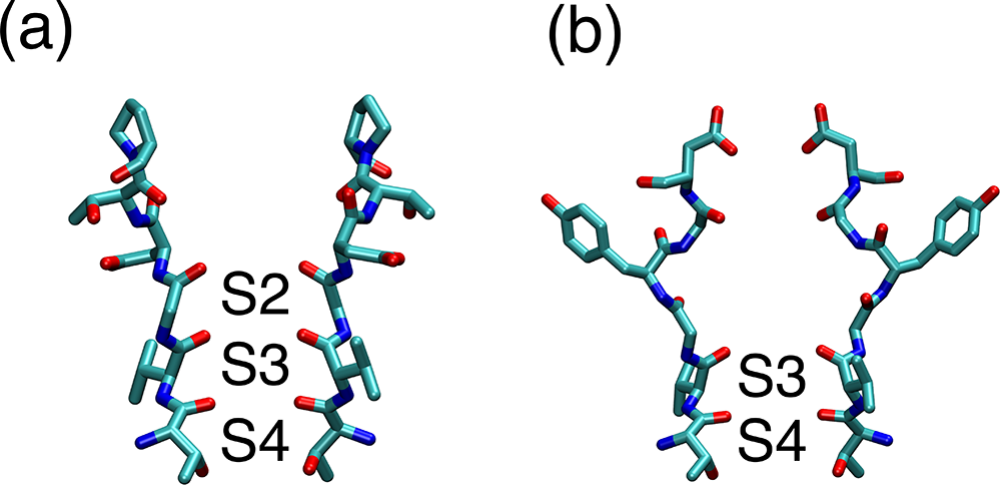
Experimental structures of the SF in NaK2CNG-D (a) and Shaker with mutation W434F (b). The residues corresponding to S624 to N629 of hERG are shown in licorice representation for two opposing subunits of the experimental structure PDB code 3K0R (a) and 7SJ1 (b). Labels are included in the binding sites that preserve the same structure observed in the conductive state.

The hypothesis that C-type inactivation in hERG is caused by an initial widening of the SF is sustained by the experimental data. Electrophysiology experiments showed that during the early stages of C-type inactivation, the hERG channel is permeable to Na^+^ in the absence of K^+^, and that only in the timescale of the order of seconds, the channel becomes completely shut to ion permeation.^38^ The closed structure of the SF observed experimentally in KcsA and in CHARMM-based MD simulations of hERG is incompatible with ion conduction.^39^ Thus, it appears unlikely that C-type inactivation of hERG is caused by an immediate closure of the SF as this could not explain the transient state permeable to sodium ions that is observed experimentally. Instead, the structure of the SF sampled in AMBER-based simulations of hERG-F627Y seems compatible with Na^+^-conduction. In hERG-F627Y, binding sites S3 and S4 are preserved, while structural changes are only recorded for binding sites S0, S1, and to some extent, S2. In terms of K^+^ binding sites, this structure of the SF resembles the experimental structure observed in NaK channels mutated to mimic the cyclic nucleotide-gated (CNG) channels,^40^ as shown in Figure 4. Electrophysiology experiments and X-ray structures demonstrated that selective conduction of K^+^ over Na^+^ in these mutated NaK channels requires the presence of four binding sites and that channels with only two or three binding sites are permeable to sodium ions,^40^ as also confirmed by energetic calculations.^41^ Thus, the structures of the SF observed in MD simulations of hERG-F627Y might represent the Na^+^ permeable state of hERG experimentally observed in the early stages of C-type inactivation. In agreement with this hypothesis, structures of the SF with only three intact binding sites were not observed in MD simulations of the non-inactivating hERG-N629D channel.

Sodium conduction across the C-type inactivated state has been experimentally observed in hERG and also in the voltage-gated Shaker channel.^42^ Furthermore, in recent experimental structures of a fast inactivating mutant of Shaker, both by cryo-EM^43^ and by X-ray crystallography,^44^ the SF appeared with binding sites S3 and S4 perfectly intact but with S1 and S2 sites enlarged compared to the canonical structure (Figure 4b). Thus, inactivation of Shaker seems to imply a widening of the extracellular portion of the SF rather than a constriction of its central region, as observed in the KcsA channel. Here, MD simulations of wild-type hERG and hERG-F627Y have also revealed a widening of the extracellular portion of the SF of the same magnitude to that observed in the experimental structure of Shaker, while binding sites S3 and S4 do not deviate from the canonical conductive state.

Since C-type inactivation is dictated by the subtle atomic interactions of the SF and ions, water molecules, and protein residues, it is not surprising that the details of C-type inactivation could be channel-dependent, and MD simulations are certainly an important tool to reveal these atomic differences among K^+^-channels. Limitations of the computational technique should be carefully considered when interpreting what it is observed in the trajectories. The shortcomings include not only the inherent approximations of force fields but also the fact that atomic models are necessarily simplified descriptions of the actual biological systems. For instance, the atomic models adopted here include only the pore region of hERG without the voltage-sensor domain. The lack of the voltage sensor is not expected to modify the structure of the pore region during the simulated timescale employed in this study; this is reflected in the stability of the intracellular gate in all the simulated trajectories starting from the open structure. The same simplification was adopted by other authors in simulations of C-type inactivation with the CHARMM force field,^12,13^ and consequently, the lack of the voltage sensor does not compromise our comparison between simulations with the two force fields. The MD simulations presented here suggest that fast C-type inactivation entails structural modification of binding sites S0 and S1 and not closure of the SF, and this may have important implications in drug discovery programs and screenings targeting the crucial hERG ion channel.

## Data and Software Availability

NAMD and VMD are available to non-commercial users under a distribution-specific license. SciPy is an open-source python library distributed under BSD license. MDAnalysis is available under the GNU General Public License, version 2. The GitHub repository https://github.com/sfurini/md_herg_ctype.git includes the atomic coordinates in the last frame of all generated trajectories (including replicas) in PDB format; topology, restart, and configuration files used for the MD simulations.

## Supporting Information Available

Diameter of the SF in simulations of the KcsA channel and diameter of the intracellular cavity in simulations of hERG, hERG-N629D, and hERG-F627Y (PDF)
