# Supplementary material for: Early Steps in C-Type Inactivation of the hERG Potassium Channel
Source: J Chem Inf Model. 2022 Dec 13;63(1):251–8. doi: 10.1021/acs.jcim.2c01028 (PMC9832476; doi:10.1021/acs.jcim.2c01028)
Supplement: Supplementary file 1 — ci2c01028_si_001.pdf [file ci2c01028_si_001.pdf]

Supporting Information

Early Steps in C-type Inactivation of the hERG Potassium Channel

Francesco Pettini[1,2], Carmen Domene[3,4], Simone Furini[5*]*

[1]Department of Medical Biotechnologies, University of Siena, viale Mario Bracci 12, Siena, 53100, Italy

[2]Department of Biotechnology, Chemistry and Pharmacy, University of Siena, viale Mario Bracci 12, Siena, 53100, Italy

[3]Department of Chemistry, University of Bath, Claverton Down, Bath, BA2 7AY, UK

[4]Department of Chemistry, University of Oxford, Mansfield Road, Oxford, OX1 3TA, UK

[5]Department of Electrical, Electronic and Information Engineering "Guglielmo Marconi", University of Bologna, via dell'Università 50, Cesena (FC), 47521, Italy

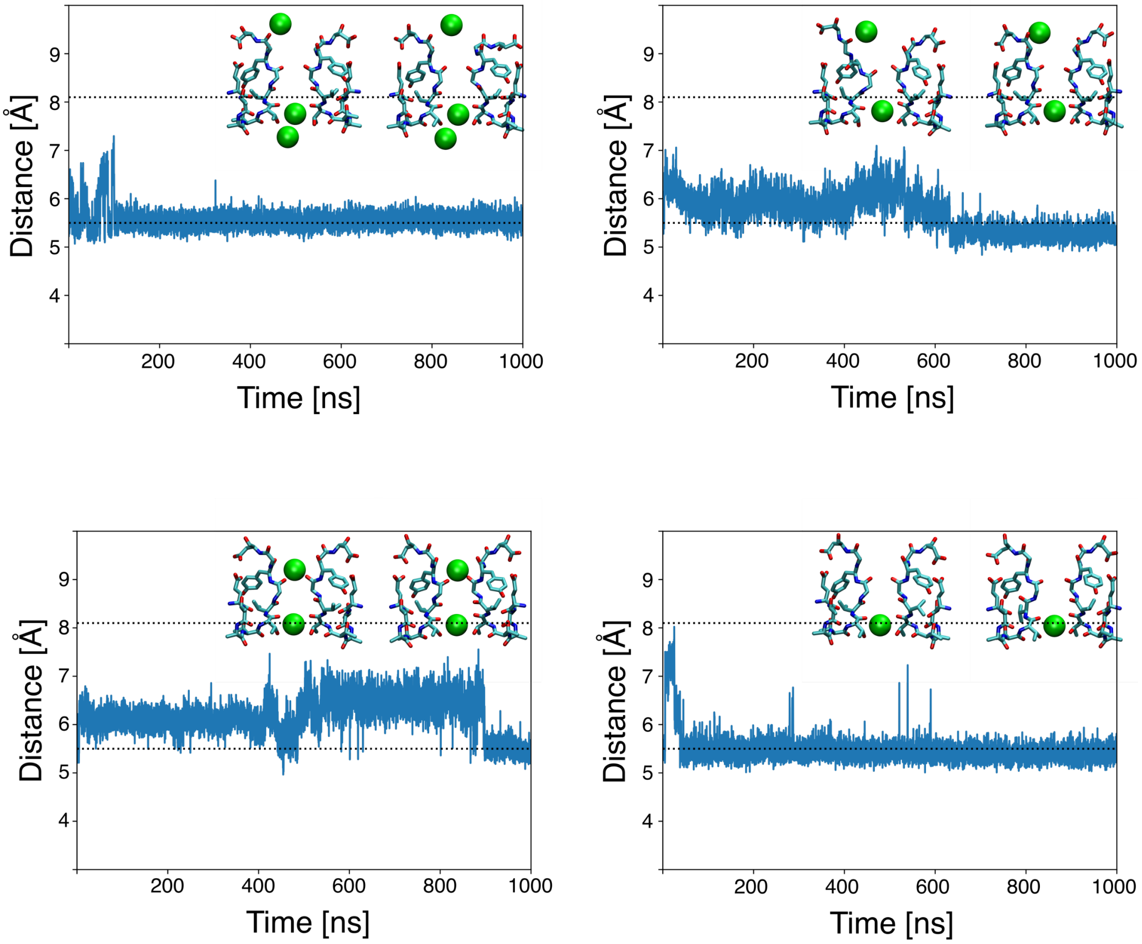

Figure S1. Diameter of the SF in simulations of KcsA. The average distance between Cα atoms of residues Gly77 in opposing subunits is shown for 4 independent replicas; this average is a contribution from two values, one from each of the two opposing subunits shown as an inset in each figure. The structures of the SF shown as insets are from the last snapshot of each trajectory, with residues Glu71 to Asp80 in licorice representation, and potassium ions as green spheres. The top and bottom dotted lines correspond to the distance between Cα atoms of residues Gly77 in the open (PDB: 1K4C) and closed (PDB:1K4D) structures respectively.

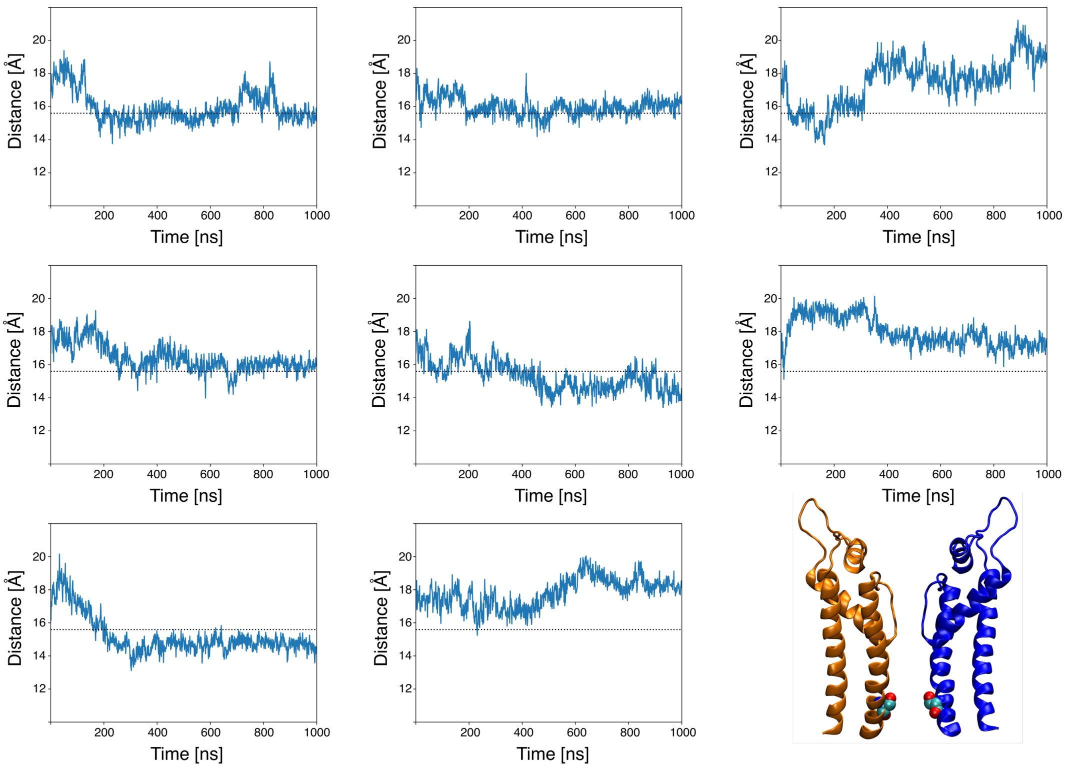

Figure S2. Diameter of the intracellular gate in simulations of hERG. The average distance between Cα atoms of residues Ser660 in opposing subunits is shown for eight independent replicas. The dotted black line corresponds to the analogous distance in the experimental structure (PDB: 5VA2). In the bottom-right corner of the figure, a snapshot of the channel is shown with only two opposing subunits, for simplicity, in cartoon representation and residues Ser660 in VdW representation.

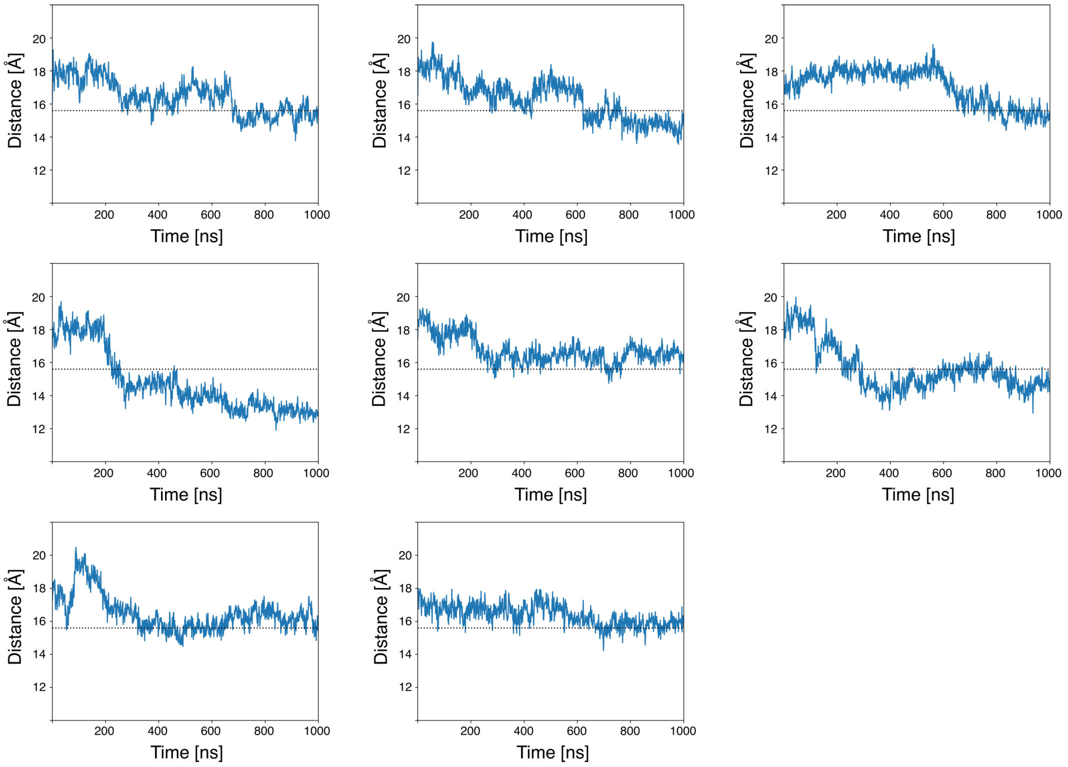

Figure S3. Diameter of the intracellular gate in simulations of hERG-N629D. The average distance between Cα atoms of residues Ser660 in opposing subunits is shown for eight independent replicas. The dotted black line corresponds to the analogous distance in the experimental structure (PDB: 5VA2).

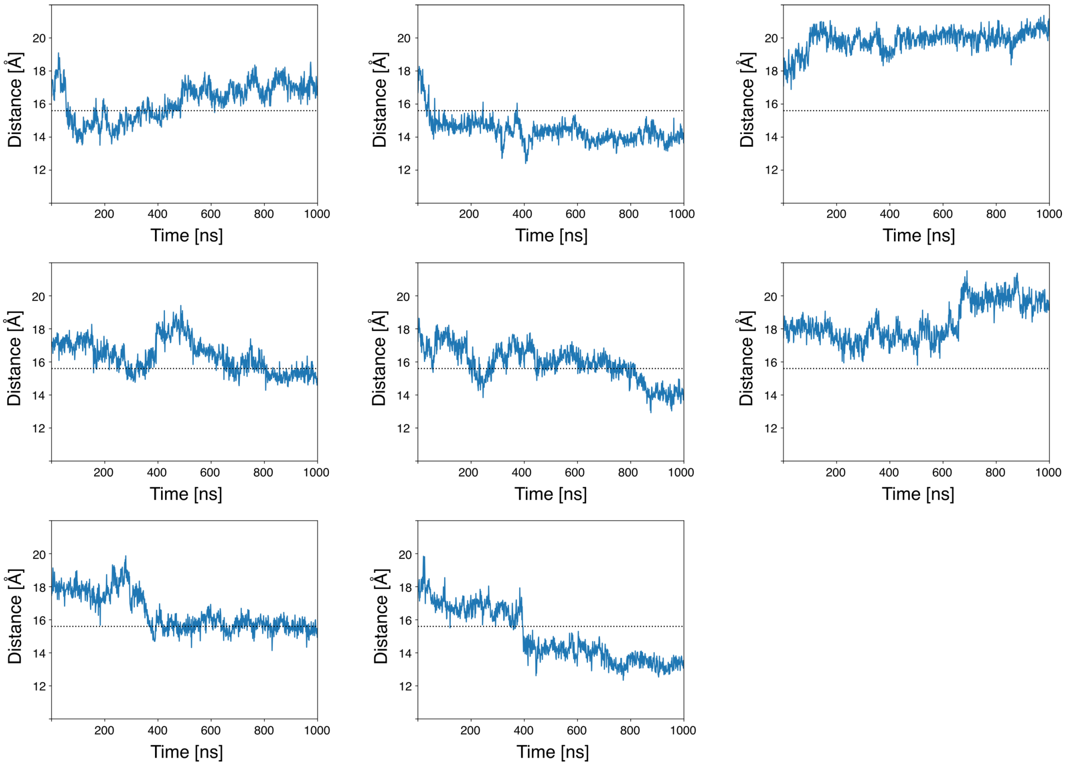

Figure S4. Diameter of the intracellular gate in simulations of hERG-F627Y. The average distance between Cα atoms of residues Ser660 in opposing subunits is shown for eight independent replicas. The dotted black line corresponds to the analogous distance in the experimental structure (PDB: 5VA2).
